# Enhanced production of 2,3-butanediol by engineered *Saccharomyces cerevisiae* through fine-tuning of pyruvate decarboxylase and NADH oxidase activities

**DOI:** 10.1186/s13068-016-0677-9

**Published:** 2016-12-09

**Authors:** Jin-Woo Kim, Jungyeon Kim, Seung-Oh Seo, Kyoung Heon Kim, Yong-Su Jin, Jin-Ho Seo

**Affiliations:** 1 Department of Agricultural Biotechnology, Center for Food and Bioconvergence, Seoul National University, Seoul, 151-921 Republic of Korea; 2 School of Life Sciences and Biotechnology, Korea University, Seoul, 136-713 Republic of Korea; 3 Department of Food Science and Human Nutrition, Institute for Genomic Biology, University of Illinois at Urbana-Champaign, Urbana, IL 61801 USA

**Keywords:** Pyruvate decarboxylase, *Saccharomyces cerevisiae*, 2, 3-Butanediol, NADH oxidase, Metabolomics, Metabolic engineering

## Abstract

**Background:**

2,3-Butanediol (2,3-BD) is a promising compound for various applications in chemical, cosmetic, and agricultural industries. Pyruvate decarboxylase (Pdc)-deficient *Saccharomyces cerevisiae* is an attractive host strain for producing 2,3-BD because a large amount of pyruvate could be shunted to 2,3-BD production instead of ethanol synthesis. However, 2,3-BD yield, productivity, and titer by engineered yeast were inferior to native bacterial producers because of the following metabolic limitations. First, the Pdc-deficient yeast showed growth defect due to a shortage of C_2_-compounds. Second, redox imbalance during the 2,3-BD production led to glycerol formation that lowered the yield.

**Results:**

To overcome these problems, the expression levels of Pdc from a Crabtree-negative yeast were optimized in *S. cerevisiae*. Specifically, *Candida tropicalis PDC1* (*CtPDC1*) was used to minimize the production of ethanol but maximize cell growth and 2,3-BD productivity. As a result, productivity of the BD5_G1CtPDC1 strain expressing an optimal level of Pdc was 2.3 folds higher than that of the control strain in flask cultivation. Through a fed-batch fermentation, 121.8 g/L 2,3-BD was produced in 80 h. NADH oxidase from *Lactococcus lactis* (*noxE*) was additionally expressed in the engineered yeast with an optimal activity of Pdc. The fed-batch fermentation with the optimized 2-stage aeration control led to production of 154.3 g/L 2,3-BD in 78 h. The overall yield of 2,3-BD was 0.404 g 2,3-BD/g glucose which corresponds to 80.7% of theoretical yield.

**Conclusions:**

A massive metabolic shift in the engineered *S. cerevisiae* (BD5_G1CtPDC1_nox) expressing NADH oxidase was observed, suggesting that redox imbalance was a major bottleneck for efficient production of 2,3-BD by engineered yeast. Maximum 2,3-BD titer in this study was close to the highest among the reported microbial production studies. The results demonstrate that resolving both C_2_-compound limitation and redox imbalance is critical to increase 2,3-BD production in the Pdc-deficient *S. cerevisiae*. Our strategy to express fine-tuned *PDC* and *noxE* could be applicable not only to 2,3-BD production, but also other chemical production systems using Pdc-deficient *S. cerevisiae*.

**Electronic supplementary material:**

The online version of this article (doi:10.1186/s13068-016-0677-9) contains supplementary material, which is available to authorized users.

## Background

2,3-Butanediol (2,3-BD) is a potential feedstock for use by the chemical, cosmetic, and agricultural industries. It is used as a precursor for synthesizing 1,3-butadiene which is a monomer for producing synthetic rubber. In addition, 2,3-BD could be used as anti-freeze agent (R,R-BD), solvents, and its derivatives could be used as flavoring agent, moisturizer, liquid fuel additive, humectant in cosmetics, and plant growth promoter [1, 2].

Many research groups have attempted producing 2,3-BD via microbial fermentations with native bacterial producers such as *Klebsiella pneumoniae*, *K. oxytoca*, *Paenibacillus polymyxa*, and *Enterobacter* species [3, 4]. These bacterial strains are able to produce 2,3-BD with high productivity, but formation of biofilm consisting of exopolysaccharides [5], optical impurity of 2,3-BD [3], and production of various by-products such as succinate, lactate, acetate, and ethanol [6] hampered the use of the strains for industrial fermentations. Commercialization is also constrained by most of 2,3-BD-producing bacteria belonging to class II (pathogenic) microorganisms, which requires tight safety regulations for industrial-scale fermentations [7]. In contrast, *Saccharomyces cerevisiae* is a GRAS (generally recognized as safe) microorganism and has been widely employed in industrial-scale fermentation processes for producing various chemicals and fuels. Thus, *S. cerevisiae* would be an appropriate microorganism for industrial production of 2,3-BD. Nonetheless, it is necessary to delete the genes coding for pyruvate decarboxylase (Pdc) for 2,3-BD production because *S. cerevisiae* produces ethanol as a major product.

Pdc-deficient *S. cerevisiae* is a promising metabolic background for producing non-ethanol products such as 2,3-BD, 3-hydroxypropionic acid, and lactic acid. It accumulates pyruvate which is a precursor of numerous chemical molecules instead of producing ethanol from glucose [8]. However, impaired growth of Pdc-deficient *S. cerevisiae* on glucose has been a major obstacle to exploit Pdc-deficient *S. cerevisiae* for 2,3-BD production. The reasons for the growth defect are (1) lack of acetyl-CoA in the cytosol [9, 10] and (2) a redox imbalance due to accumulation of cytosolic NADH [11, 12]. Cytosolic acetyl-CoA is indispensable for growth of *S. cerevisiae* because it is used for synthesizing lysine and fatty acids in the cytosol [8–10]. Pdc-deficient *S. cerevisiae* cannot synthesize cytosolic acetyl-CoA from glucose because the deletion of PDC leads to elimination of cytosolic C_2_-compounds (e.g., acetaldehyde, acetate, ethanol). Within mitochondria, the pyruvate dehydrogenase (Pdh*)* complex converts pyruvate into acetyl-CoA, but mitochondrial acetyl-CoA cannot pass through the inner membrane of mitochondria [13]. Although the YBR219C and YBR220C are known as putative genes coding for acetyl-CoA transporter, activities of these enzymes were not sufficient for supplying enough acetyl-CoA to cytosol [14]. Therefore, cell growth and carbon utilization of Pdc-deficient *S. cerevisiae* strains were greatly inhibited by insufficient supply of cytosolic acetyl-CoA, which is often termed as ‘C_2_-auxotrophy’. Redox imbalance is another reason for growth defect of Pdc-deficient *S. cerevisiae* on glucose. Excess NADH is generated in Pdc-deficient *S. cerevisiae* because oxidation of cytosolic NADH via the ethanol production pathway is blocked. NADH generated by converting glucose to pyruvate should be re-oxidized to NAD^+^ to maintain cellular redox metabolism. However, insufficient activity of the respiratory pathway because of the glucose-induced Crabtree effect [11] and absence of transhydrogenase activity [15, 16] aggravate the redox imbalance of Pdc-deficient *S. cerevisiae*.

There have been several reports about the production of 2,3-BD with engineered *S. cerevisiae* harboring the bacterial 2,3-BD biosynthetic enzymes [12, 17, 18]. By introduction of *B. subtilis* α-acetolactate synthase (*alsS*) and α-acetolactate decarboxylase (*alsD*), and overexpression of endogenous 2,3-butanediol dehydrogenase (*BDH1*), pyruvate was converted into optically pure (2R,3R)-BD by the Pdc-deficient *S. cerevisiae* with *MTH1* mutation [12].

The mutation (G241C) in *MTH1* has been reported to suppress the growth defect of the Pdc-deficient *S. cerevisiae* strain [12]. In the presence of extracellular glucose, signal transduction via the glucose sensors (Rgt2/Snf3) and casein kinases (Yck1/2) induces phosphorylation of Mth1 to be degraded [19]. The degradation of Mth1 led to the down-regulation of hexose transporter genes (*HXT*s) [20], resulting in decreased glucose influx rate in spite of extracellular glucose [21]. A slow glucose uptake rate caused by the *MTH1* mutation might be responsible for restoration of growth defect by the Pdc-deficient strains on glucose [12]. Additionally, although the exact mechanism remains unknown, the mutant *MTH1* could partially relieve the C_2_-auxotrophy of Pdc-deficient *S. cerevisiae* [12, 21]. The mutation in *MTH1* might be regarded as an indispensable strategy for Pdc-deficient *S. cerevisiae* to grow on glucose, but the slow glucose consumption rates caused by the *MTH1* mutation resulted in much lower 2,3-BD productivity [12] than that by bacterial 2,3-BD production systems [22, 23]. The lower glucose consumption rates and 2,3-BD productivity by the Pdc-deficient *S. cerevisiae* are putatively caused by reduced expression levels of *HXTs* by *MTH1* mutation [19, 20, 24]. As such, metabolic engineering strategies to alleviate the growth defect of Pdc-deficient *S. cerevisiae* without mutations in *MTH1* needs to be devised for efficient and rapid production of 2,3-BD by engineered yeast.

In this study, we present metabolic engineering strategies for rapid production of 2,3-BD by the Pdc-deficient *S. cerevisiae* strain without mutating *MTH1*. To this end, we attempted to address both C_2_-auxotrophy and redox imbalance. For the construction of C_2_-independent strains, various *PDC* genes from the Crabtree-negative yeast were expressed at a minimal level to eliminate the C_2_-auxotrophy of Pdc-deficient *S. cerevisiae* strains. As excess expression of *PDC* produces a large amount of ethanol instead of producing 2,3-BD, we hypothesized that expression levels of *PDC* need to be fine-tuned to balance metabolic fluxes between acetyl-CoA synthesis and 2,3-BD production. Specifically, *PDC* expression levels were optimized through the variations of *PDC* gene sources, promoters, and copy numbers. Additionally, *Lactococcus lactis* NADH oxidase (*noxE*) was co-expressed to resolve the issue of redox imbalance [18]. The resulting engineered *S. cerevisiae* strain expressing both PDC and *noxE* at optimal levels produced 2,3-BD with much higher titer, productivity, and yield than those of the other engineered yeast capable of producing 2,3-BD. The results presented in this study suggest that utilization of the engineered *S. cerevisiae* strain for 2,3-BD production could be an attractive alternative to bacterial 2,3-BD production systems [25].

## Results

### A mutation in *MTH1* decreased glucose uptake rate in Pdc-deficient *S. cerevisiae*

The mutations in *MTH1* were identified from evolved strains exhibiting improved growth phenotypes of Pdc-deficient *S. cerevisiae*. In particular, a mutation (G241C) was employed for improving 2,3-BD production by the *S. cerevisiae* strain because the *MTH1* mutation could overcome the C_2_-auxotrophy of Pdc-deficient *S. cerevisiae* [12, 21]. However, the *MTH1* mutation resulted in reduced expression levels of hexose transporters in *S. cerevisiae* [11, 20], causing slow glucose uptake rates. Batch fermentations were performed for comparing 2,3-BD production by 2,3-BD-producing strains with the *MTH1* mutation (the BD4 strain) and without the *MTH1* mutation (the BD5 strain) (Additional file 1: Fig. S1). When glucose was used as a sole carbon source (Additional file 1: Fig. S1A, B), the maximum dry cell weight (DCW) of the BD4 strain was 5.5-fold higher (2.2 g/L) than that of the BD5 strain (0.4 g/L). The BD4 strain (with the *MTH1* mutation) showed higher volumetric glucose uptake rate and 2,3-BD productivity (0.289 g glucose/L·h and 0.087 g 2,3-BD/L·h) than those (0.155 g glucose/L·h and 0.051 g 2,3-BD/L·h) of the BD5 strain (without the *MTH1* mutation). However, when a small amount of ethanol (0.5 g/L) was supplemented as a C_2_-compound (Additional file 1: Fig. S1C, D), the maximum DCW of the both strains were similar (3.9 and 4.2 g/L), but the BD5 strain (without the MTH1 mutation) showed a much higher volumetric glucose uptake rate and 2,3-BD productivity (1.23 g glucose/L·h and 0.305 g 2,3-BD/L·h) than the BD4 strain (0.665 g glucose/L·h and 0.205 g 2,3-BD/L·h). Thus, we concluded that the BD5 strain without the *MTH1* mutation can grow and consume glucose with comparable to the wild type and produce 2,3-BD efficiently as long as C_2_-compounds are properly supplied.

### Optimization of *PDC* expression levels led to enhanced 2,3-BD production through relieving C_2_-auxotrophy in Pdc-deficient *S. cerevisiae*

Partial restoration of Pdc might be a simple way to supply a C_2_-compound for relieving the C_2_-auxotrophy in Pdc-deficient *S. cerevisiae*. As shown in Additional file 1: Fig. S1, the addition of only a small amount of ethanol (0.5 g/L) greatly enhanced 2,3-BD productivity in the BD5 strain. This result suggests that fine-tuning of Pdc activity possibly maximizes 2,3-BD productivity, while keeping the 2,3-BD yield unchanged by minimizing ethanol production. Therefore, a set of *PDC* expression cassettes with different *PDC* gene sources, promoters, and copy numbers was constructed to identify optimal levels of Pdc for enhanced 2,3-BD production without introducing *MTH1* mutations. First, we searched for the Pdc enzymes with low activities in *S. cerevisiae*. As Crabtree-negative yeasts exhibit lower Pdc activities than Crabtree-positive yeasts [26–28], we reasoned that Pdc activities might be maintained at low levels even under excess glucose conditions when the *PDC* genes from Crabtree-negative yeasts such as *C. tropicalis* and *K. marxianus* [26, 28] are optimally expressed. The kinetic parameters of *C. tropicalis* Pdc1 (CtPdc1), *K. marxianus* Pdc1, and endogenous Pdc1, Pdc5, and Pdc6 were examined after expressing each of the genes in the Pdc-deficient *S. cerevisiae* strain (Additional file 1: Table S1). As CtPdc1 showed the lowest *V*_max_ value among the five Pdc enzymes, the *CtPDC1* gene was selected to confer Pdc activity in the 2,3-BD-producing strain. The comparison of amino acid sequences of CtPdc1 and ScPdc1 was depicted in Additional file 1: Fig. S2. The Ctpdc1 and Scpdc1 proteins have 66% identical amino acid residues. Both Ctpdc1 and Scpdc1 proteins retain all structurally important amino acid residues for the Mg^2+^ and thiamine pyrophosphate (ThDP) binding loop (N473, N474, G476, D446, T392, S448, I479) and substrate activation (C223) that are conserved throughout all *PDC*s [29–31]. In order to identify an optimal expression level of *CtPDC1* for maximizing 2,3-BD production and minimizing ethanol production, the *CtPDC1* gene under the control of various promoters in plasmids with different copy numbers was expressed in the BD5 strain (Table 1). Then, in vitro Pdc activities in the control and Pdc-expressed strains were measured (Additional file 1: Fig. S3). As expected, each strain exhibited different levels of Pdc activities. The Pdc activities of the BDt_C1CtPDC1, BD5_G1CtPDC1, BD5_C2CtPDC1, and BD5_T2CtPDC1 strains were 74.7, 122.1, 287.4, and 1387.0 mU/mg protein, respectively.

**Table 1:** Strains and plasmids used in this study

Strains and plasmids	Description	Reference
Strains		
*Candida tropicalis* ATCC20336	Source for *CtPDC1*	In this study
*Kluyveromyces marxianus* KCTC17555	Source for *KmPDC1*	In this study
*Saccharomyces cerevisiae* D452-2	Source for *ScPDC1*, *ScPDC5, ScPDC6 MATα leu2 his3 ura3*	[49]
BD4	D452-2, *pdc1Δ, pdc5Δ,* evolved 2,3-BD-producing strain	[12]
SOS5	D452-2, *pdc1Δ, pdc5Δ, pdc6Δ*	[18]
SOS5_T2CtPDC1	SOS5, p426TDH3_CtPDC1	In this study
SOS5_T2KmPDC1	SOS5, p426TDH3_KmPDC1	In this study
SOS5_T2ScPDC1	SOS5, p426TDH3_ScPDC1	In this study
SOS5_T2ScPDC5	SOS5, p426TDH3_ScPDC5	In this study
SOS5_T2ScPDC6	SOS5, p426TDH3_ScPDC6	In this study
BD5	SOS5, p423_alsSalsD, p425_BDH1	[18]
BD5_Con	BD5, p426GPD	[18]
BD5_C1CtPDC1	BD5, p406CYC1_CtPDC1	In this study
BD5_G1CtPDC1	BD5, p406GPD2_CtPDC1	In this study
BD5_C2CtPDC1	BD5, p426CYC1_CtPDC1	In this study
BD5_T2CtPDC1	BD5, p426TDH3_CtPDC1	In this study
BD5_G1CtPDC1_nox	BD5, p406GPD2_CtPDC1, pAUR_Llnox	In this study
Plasmids		
pRS406	*URA3*	[50]
pRS426	*URA3* 2 μm origin	[50]
p406GPD2	pRS406, *GPD2*_prom_ *CYC1*_term_	[18]
p426CYC1	pRS426, *CYC1*_prom_ *CYC1*_term_	[18]
p426GPD	*URA3* 2 μm origin, *TDH3*_prom_ *CYC1*_term_	[50]
p423_alsSalsD	*HIS3* 2 μm origin, *TDH3*_prom_-*alsS*-*CYC1*_term_ *TDH3*_prom_-*alsD*-*CYC1*_term_	[51]
p425_BDH1	*LEU2* 2 μm origin, *TDH3*_prom_-*BDH1*-*CYC1*_term_	[12]
p426TDH3_Llnox	pRS426, *TDH3*_prom_-*Llnox*-*CYC1*_term_	[18]
p426TDH3_CtPDC1	pRS426, *TDH3*_prom_-*CtPDC1*-*CYC1*_term_	In this study
p426TDH3_KmPDC1	pRS426, *TDH3*_prom_-*KmPDC1*-*CYC1*_term_	In this study
p426TDH3_ScPDC1	pRS426, *TDH3*_prom_-*ScPDC1*-*CYC1*_term_	In this study
p426TDH3_ScPDC5	pRS426, *TDH3*_prom_-*ScPDC5*-*CYC1*_term_	In this study
p426TDH3_ScPDC6	pRS426, *TDH3*_prom_-*ScPDC6*-*CYC1*_term_	In this study
p406CYC1_CtPDC1	pRS406, *CYC1*_prom_-*CtPDC1*-*CYC1*_term_	In this study
p406GPD2_CtPDC1	pRS406, *GPD2*_prom_-*CtPDC1*-*CYC1*_term_	In this study
p426CYC1_CtPDC1	pRS426, *CYC1*_prom_-*CtPDC1*-*CYC1*_term_	In this study
pAUR_Llnox	*AUR1*-*C CEN6 ARS4*, *TDH3*_prom_-*Llnox*-*CYC1*_term_	In this study

**Fig. 1:**
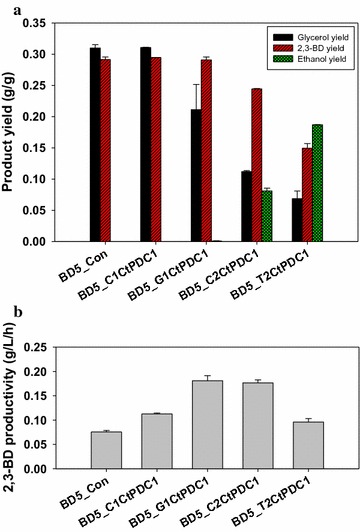
Comparison of **a** Glycerol, 2,3-BD, and ethanol yields, and **b** 2,3-BD productivity in batch fermentation for the control and four engineered strains expressing *CtPDC1* gene with different expression levels. Values are calculated from experiments in Additional file 1: Figure S5. Results are the averages of duplicate experiments and *error bars* indicate standard deviation

**Fig. 2:**
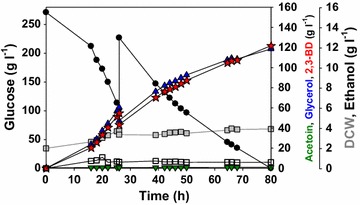
Fed-batch fermentation profiles of the BD5_G1CtPDC1 strain in YP medium with bioreactor. Symbols: Glucose (*filled circle*), dry cell weight (*gray square*), glycerol (*blue triangle*), acetoin (*green inverted triangle*), 2,3-BD (*red star*), and ethanol (*open square*)

To evaluate the effects of different Pdc activities on 2,3-BD production by the engineered yeast, batch fermentations were carried out in minimal medium containing 90 g/L glucose (Fig. 1; Additional file 1: Fig. S4). The maximum DCW and glucose consumption rate increased after the expression of *CtPDC1*. The maximum DCW (1.04 ± 0.06 g/L) of the BD5_G1CtPDC1 strain (*CtPDC1* under *GPD* promoter and chromosomal integration) which has moderate Pdc activity among the engineered strains was 3.5-fold higher than the control strain (0.30 ± 0.00 g/L). While the BD5_G1CtPDC1 strain consumed 74.6 ± 3.1 g/L glucose in 120 h cultivation, the control strain consumed only 31.2 ± 1.6 g/L glucose during the same time period. The engineered strains showed different product profiles according to the *PDC* expression levels. In contrast to the BD5_T2CtPDC1 (*CtPDC1* under the *TDH3* promoter in a multi-copy plasmid) strain that produced a large amount of ethanol, the BD5_G1CtPDC1 strain produced less than 0.5 g/L ethanol and exhibited the highest productivity among the engineered 2,3-BD-producing strains. The 2,3-BD productivity of the BD5_G1CtPDC1 strain (0.18 ± 0.01 g 2,3-BD/L·h) was 2.3-fold higher than that of the BD5_Con strain (0.08 ± 0.00 g 2,3-BD/L·h). When fed-batch fermentation of the BD5_G1CtPDC1 strain was performed, 121.8 g/L of 2,3-BD was produced (Fig. 2). The volumetric productivity of 2,3-BD production reached 1.52 g 2,3-BD/L·h which is 3.9-fold higher than the case with the BD4 strain (0.39 g 2,3-BD/L·h) in a previous report [12]. Small amounts (<1.0 g/L) of ethanol and acetoin accumulated, but a large amount of glycerol (118.5 g/L) was produced as a by-product. A yield of glycerol (0.333 g glycerol/g glucose) was similar to that of 2,3-BD (0.337 g 2,3-BD/g glucose). These results suggest that optimal expression of *CtPDC1* can increase glucose uptake and 2,3-BD production rates substantially, but aggravate the yield of 2,3-BD production due to massive glycerol production.

### Expression of NADH oxidase increased 2,3-BD yield and productivity

**Fig. 3:**
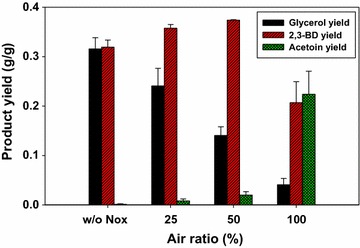
Glycerol, 2,3-BD, and acetoin yields of the BD5_G1CtPDC1 strain (w/o Nox), and the BD5_G1CtPDC1_nox strain (25, 50, 100) in batch cultivation with bioreactor. Aeration conditions were controlled by ratios of mixed inlet gas at constant agitation speed of 500 rpm and air flow rate of 2 vvm: only air (w/o Nox and 100), 1:1 mixture of air and nitrogen gas (50), 1:3 mixture of air and nitrogen gas (25)

We reasoned that glycerol production during the 2,3-BD production is due to surplus NADH production in the cytosol as the 2,3-BD-producing pathway is not redox-neutral [18]. Excess NADH in the cytosol should be oxidized in order to reduce glycerol formation and increase 2,3-BD yield. It has been reported that the expression of *L. lactis* NADH oxidase in *S. cerevisiae* suppressed glycerol formation by reducing intracellular NADH/NAD^+^ levels [18]. As such, *L. lactis* NADH oxidase was additionally expressed in the BD5_G1CtPDC1 strain. Because molecular oxygen acts as a substrate for the NADH oxidase reaction, aeration conditions could influence the in vivo activity of NADH oxidase. In order to evaluate the effects of NADH oxidase expression on 2,3-BD production, the batch fermentations with the BD5_G1CtPDC1 and BD5_G1CtPDC1_nox strains were performed under low (25%), medium (50%), and high (100%) aeration conditions. As expected, the yields of 2,3-BD and glycerol significantly changed according to the levels of aeration (Fig. 3). Under the high aeration condition, the glycerol yield of the BD5_ G1CtPDC1_nox strain (0.041 ± 0.013 g glycerol/g glucose) was only 12.3% of that of the BD5_G1CtPDC1 strain (0.316 ± 0.022 g glycerol/g glucose). Interestingly, a large amount of acetoin (0.224 ± 0.047 g acetoin/g glucose) was produced by BD5_ G1CtPDC1_nox under the high aeration condition, suggesting that surplus NADH was oxidized by NADH oxidase and generated an NADH-deficient condition. The highest 2,3-BD yield (0.374 ± 0.001 g 2,3-BD/g glucose) was observed under the medium aeration condition. The influence of in vivo activity of NADH oxidase on NADH and NAD^+^ concentrations in the BD5_ G1CtPDC1_nox strain was analyzed (Additional file 1: Fig. S5). While the NADH/NAD^+^ ratio (0.74) under the low aeration condition was similar to that under the medium aeration condition (0.72), the NADH/NAD^+^ ratio under the high aeration condition decreased substantially (0.45).

### In vivo activity of NADH oxidase influence on intracellular metabolites

Our results from NADH oxidase expression in the Pdc-deficient strain suggested that cofactor imbalance induced by the deletion of *PDC* genes in *S. cerevisiae* could be relieved by the expression of NADH oxidase and control of aeration conditions. To investigate the effects of NADH oxidase at metabolite levels, intracellular metabolites were analyzed from batch fermentations with the BD5_G1CtPDC1 and BD5_G1CtPDC1_nox strains. The batch fermentations were carried out under different conditions including aerobic cultivation of the BD5_G1CtPDC1 strain (Ct100), and microaerobic (N50) and aerobic (N100) cultivation of the BD5_G1CtPDC1_nox strain (Additional file 1: Fig. S6). Additional expression of NADH oxidase led to reduced glycerol yields and increased acetoin yields regardless of aeration conditions. Moreover, glucose consumption rate (2.68 g glucose/L·h) of the Ct100 condition increased to 4.35 g glucose/L·h for the N50 condition and 3.98 g glucose/L·h for the N100 condition. Maximum DCWs (2.5 g cell/L) under the Ct100 and N50 conditions were similar, but it increased to 3.9 g/L under the N100 condition.

From the BD5_G1CtPDC1 and BD5_ G1CtPDC1_nox cells harvested at 22 h, 129 intracellular metabolites were identified and quantified (Additional file 1: Table S2). These metabolites belonged to different chemical classes including amino acids, organic acids, sugars and sugar alcohols, fatty acids, phosphates, amines, nucleotides. Multivariate data were analyzed by principal component analysis (PCA) (Additional file 1: Fig. S7) and hierarchical clustering analysis (HCA) (Additional file 1: Fig. S8) to explore the variations of intracellular metabolites in response to NADH oxidase expression. As shown in Additional file 1: Fig. S7 and Additional file 1: Fig. S8, the samples from each condition (Ct100, N50, and N100) were clearly distinguished. It indicated that in vivo activity of NADH oxidase changed intracellular metabolism and displayed distinctive metabolic characteristics. The differences in intracellular metabolites of glycolysis, TCA cycle, and amino acid synthesis between Ct100 and N50 conditions were depicted in Additional file 1: Fig. S9. Intermediate metabolites in the glycolytic pathway including glucose, glucose-6-phosphate, fructose-6-phosphate, and pyruvate, and in the TCA cycle including isocitrate and fumarate significantly increased in Ct100 condition compared to N50 condition.

### Fed-batch fermentation with the engineered strain co-expressing *CtPDC1* and *noxE*

**Fig. 4:**
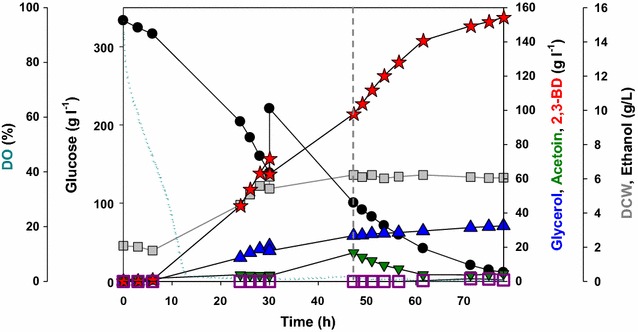
Fed-batch fermentation profiles of the BD5_G1CtPDC1_nox strain in YP medium with bioreactor. The *dashed line* represents a shift of aeration condition from 500 rpm/2 vvm to 300 rpm/1 vvm. Symbols: DO (*dot*), glucose (*filled circle*), dry cell weight (*gray square*), glycerol (*blue triangle*), acetoin (*green inverted triangle*), 2,3-BD (*red star*), and ethanol (*open square*)

A fed-batch fermentation was carried out to examine whether the BD5_ G1CtPDC1_nox strain can be a promising strain to produce 2,3-BD (Fig. 4). Aeration conditions were controlled in two stages to maximize 2,3-BD and minimize by-products formation according to the batch fermentation results shown in Fig. 3. In the first stage, fermentation medium was fully aerated for suppressing glycerol formation. As a result, 97.9 g/L of 2,3-BD and 16.7 g/L of acetoin were produced at 42.3 h. In the second stage where oxygen-limited conditions were maintained, the produced acetoin was converted into 2,3-BD. Finally, 154.3 g/L of 2,3-BD was produced with an impressive volumetric productivity of 1.98 g 2,3-BD/L/h. The overall yield of 2,3-BD was 0.404 g 2,3BD/g glucose which is 80.7% of the theoretical maximum yield. Glycerol yield (0.088 g glycerol/g glucose) decreased by 73.6% and final DCW (6.1 g/L) increased by a 1.6-fold as compared with those of the BD5_G1CtPDC1 strain in fed-batch fermentation (Fig. 2). Acetate was produced after 30 h, and the final acetate concentration reached 2.3 g acetate/L.

## Discussion

It has been known that Pdc-deficient *S. cerevisiae* cannot grow on glucose as a sole carbon source because of C_2_-auxotrophy and redox imbalance in the cytosol [10]. However, as shown in Additional file 1: Fig. S1B, the engineered strain with the 2,3-BD pathway grew slowly in synthetic medium with glucose as a sole carbon source. The growth recovery of Pdc-deficient *S. cerevisiae* by introduction of the 2,3-BD pathway was also observed in the other studies [32]. In the engineered strains harboring the 2,3-BD biosynthetic genes, C_2_-compounds might be supplied by α-acetolactate synthase (AlsS) which has a decarboxylase activity on pyruvate [33]. In addition, redox imbalance could be relieved by the 2,3-BD biosynthetic pathway because NADH could be additionally oxidized by producing 2,3-BD from pyruvate. However, the growth recovery by introduction of the 2,3-BD pathway is still insufficient for efficient production of 2,3-BD, and hence additional metabolic engineering is needed for enhancing growth rate, 2,3-BD yield, and productivity.

In other studies, the use of the *MTH1* variant has been regarded as an efficient way to overcome the growth defect of Pdc-deficient *S. cerevisiae* on glucose [12, 21]. The specific growth rate was substantially increased by the mutation on the *MTH1* gene (ref, Additional file 1: Fig. S1). However, suppressed expression of hexose transporters by mutation on the *MTH1* gene [34, 35] became another problem for the efficient production of 2,3-BD. The specific glucose uptake rate of the BD4 strain harboring the *MTH1* mutation was only 20% that of the BD5 strain with the wild-type *MTH1* gene (Additional file 1: Fig. S10). As a result, the volumetric productivity of the BD5 strain was much higher than that of the BD4 strain when the growth rate of the BD5 strain was enhanced by supplementation of ethanol as a C2-compound (Additional file 1: Fig. S1C, D). Therefore, we constructed the engineered Pdc-deficient *S. cerevisiae* strain to overcome C_2_-auxotrophy and redox balance without *MTH1* mutation.

As shown in Fig. 1, the product yields and 2,3-BD productivities were changed according to the levels of Pdc activity. The BD5_Con strain produced 2,3-BD dominantly, but ethanol production was positively correlated with Pdc activity. The BD5_G1CtPDC1 strain was selected as the best 2,3-BD production strain because ethanol production was minimized along with the sufficient supplementation of a C_2_-compound for cell growth. The production of ethanol by additional *PDC* expression led to reduced glycerol production because cofactor imbalance might be relieved by ethanol biosynthesis which is a redox-neutral process [36]. As a result, through fine-tuning expression levels of Pdc, C_2_-compounds were efficiently supplied for synthesizing acetyl-CoA and 2,3-BD or ethanol production could be easily modulated.

Expression of NADH oxidase in 2,3-BD-producing Pdc-deficient *S. cerevisiae* reduced glycerol production as a by-product by relieving cofactor balance in the cytosol (Fig. 3). *L. lactis* NADH oxidase is an H_2_O-forming NADH oxidase involved in the oxygen defense mechanism in facultative anaerobic bacteria [37]. Several studies about the effect of water-forming NADH oxidase in *S. cerevisiae* strains have been reported [18, 38–40]. Especially, *L. lactis* NADH oxidase has a lower Km value (4.1 µM) for NADH [41] than other metabolic enzymes such as Gpd (23 µM) and Bdh (55 µM) [42, 43], the expression of NADH oxidase caused a large change in metabolic fluxes. The expression of NADH oxidase in *S. cerevisiae* reduced overflow metabolism [38] and decreased production of reduced metabolites such as ethanol, succinate, 2-hydroxyglutarate, glycerol, and xylitol [39, 40]. In some cases, the introduction of NADH oxidase led to reduced biomass formation because of decreasing the NADH pool and accumulating acetaldehyde at toxic levels [38, 40]. However, introduction of NADH oxidase efficiently increased 2,3-BD yield and productivity by relieving cofactor imbalance. The possible reasons for this difference are as follows. First, Pdc-deficient *S. cerevisiae* with the 2,3-BD production pathway produces excess NADH in the cytosol. Two moles of NADH were generated by converting one mole of glucose into two moles of pyruvate, but each mole of NADH could be oxidized by producing one mole of 2,3-BD from two moles of pyruvate. The depletion of cytosolic NADH by NADH oxidase might be prevented because the excess cytosolic NADH was generated in the engineered strain. Second, as shown in Fig. 3 and Additional file 1: Fig. S5, the in vivo activity of NADH oxidase could be controlled by aeration condition. As the cells expressing *L. lactis* NADH oxidase had a high demand for dissolved oxygen (DO) [40] and rapidly reduced DO levels in fed-batch fermentation [18], the oxygen-limited cultivation could be carried out regardless of aerobic condition. Thus, the optimized aeration condition was able to efficiently enhance 2,3-BD yield and productivity and to reduce by-product formation (Fig. 4). Third, in fully aerated condition, acetoin which is the oxidized form of 2,3-BD was over-produced by the 2,3-BD-producing Pdc-deficient *S. cerevisiae* strain. Acetoin exerts no deleterious effect in contrast to acetaldehyde, which can inhibit growth and carbon utilization at concentrations above 1.0 g/L [44]. In addition, acetoin could be converted into 2,3-BD under oxygen-limited condition (Fig. 4). Thus, acetoin could be acted as a redox sink in the engineered strain for maintaining redox balance that could be induced by excessive NADH oxidase activity.

Interestingly, the expression of NADH oxidase increased glucose uptake rate as well as altered product yields. Similarly, the presence of NADH oxidase or alternative oxidase improved specific glucose uptake rate and subsequent metabolism in the wild-type background [38]. The metabolomic analysis led to a possible hypothesis for this. The concentrations of glucose, and glucose-6-phosphate, and pyruvate (intermediates of glycolysis) increased in the BD5_G1CtPDC1 strain (Ct100) compared with the BD5_G1CtPDC1_nox strain (N50). Pyruvate formation by glycolysis is NADH-generating process. Since cofactor imbalance by reduced oxidation of cytosolic NADH inhibited glycolytic flux and cell growth rate in Pdc-deficient *S. cerevisiae* [12, 21], the inhibition of glycolytic flux in the BD5_G1CtPDC1 strain might reduce the accumulation of glycolytic intermediates. Metabolic reactions with isocitrate and fumarate as substrate are also NADH-generating steps in the TCA cycle. Similar to glycolytic intermediates, these compounds might accumulate because of intracellular cofactor imbalance of NADH in the BD5_G1CtPDC1 strain (Ct100). From the comparison of intracellular metabolites in the BD5_G1CtPDC1 and BD5_G1CtPDC1_nox strains, the relieved cofactor imbalance by NADH oxidase changed glycolytic and TCA cycle intermediates. These data could explain the improved glucose consumption rate by the expression of NADH oxidase in Pdc-deficient *S. cerevisiae*.

## Conclusions

In this study, a supplementation of a C_2_-compound by optimized expression of Pdc and relieved cofactor imbalance by controlled in vivo activity of NADH oxidase enhanced 2,3-BD yield, productivity, and titer in 2,3-BD-producing Pdc-deficient *S. cerevisiae*. The 2,3-BD productivity was increased fivefold compared to the previous report [12]. In addition, final titer of 2,3-BD (154.3 g/L) is one of the highest concentration reported for production of 2,3-BD by engineered yeasts and bacterial native producers. To improve 2,3-BD production performance by the engineered yeast strains, efficient utilization of various sugars such as galactose, xylose, and cellobiose or elimination of glycerol formation are required. In addition, optimizing aeration condition is important in large-scale fermentations. The fine-tuned expression of *PDC* and co-expression of NADH oxidase in Pdc-deficient *S. cerevisiae* could be applied to improving other chemical production systems using pyruvate as a precursor compound.

## Methods

### Construction of plasmids

Strains and plasmids used in this study are summarized in Table 1. The primers used for cloning of the *PDC* genes from various yeast species and *AUR1*-*C* gene are listed in Table 2. *E. coli* TOP10 (Invitrogen, Carlsbad, CA) was used for gene cloning and manipulation. *E. coli* transformants were grown in Lysogeny Broth (LB) medium with 100 μg/mL of ampicillin. To construct expression plasmids with different *PDC* genes, ORFs of *PDC* genes were amplified by PCR from genomic DNA of *C. tropicalis*, *Kluyveromyces marxianus*, and *S. cerevisiae* using the primers in Table 2. The amplified DNA fragments were ligated into appropriate restriction sites in p426GPD, p406CYC1, and p406GPD2 plasmids. In order to construct aureobasidin A resistance plasmids, the *AUR1*-*C* gene was amplified from pAUR101 plasmids by PCR with primers of F_SnaBI_AUR1-C and R_MfeI_AUR1-C. The amplified DNA fragments were ligated into the corresponding restriction sites in Table 2.

**Table 2:** Primers used in this study

Primers	Restriction site	Sequence
Cloning of PDC genes		
F_XmaI_CtPDC1	***Xma*** **I**	tccc**CCCGGG**aaaatgtctgaaattactttgggtag
R_SalI_CtPDC1	***Sal*** **I**	acgc**GTCGAC**tttattcttgagcagcgttg
F_XmaI_KmPDC1	***Xma*** **I**	tccc**CCCGGG**aaaatgtctgaaattactctaggtcg
R_SalI_KmPDC1	***Sal*** **I**	acgc**GTCGAC**tttattcttgcttggcgtt
F_XmaI_ScPDC1	***Xma*** **I**	tccc**CCCGGG**atgtctgaaattactttgggtaa
R_SalI_ScPDC1	***Sal*** **I**	acgc**GTCGAC**ttattgcttagcgttggtag
F_XmaI_ScPDC5	***Xma*** **I**	tccc**CCCGGG**atgtctgaaataaccttaggtaaata
R_SalI_ScPDC5	***Sal*** **I**	acgc**GTCGAC**ttattgtttagcgttagtagcg
F_XmaI_ScPDC6	***Xma*** **I**	tccc**CCCGGG**atgtctgaaattactcttggaaaatac
R_SalI_ScPDC6	***Sal*** **I**	acgc**GTCGAC**ttattgtttggcatttgtagc
Cloning of *AUR1*-*C* gene		
F_SnaBI_AUR1-C	***SnaB*** **I**	agcttgtcacct**TACGTA**aaagtgcccatcagtgttc
R_MfeI_AUR1-C	***Mfe*** **I**	ataaccgggt**CAATTG**cagaggaaagaataacgcaa

Bold and capital characters are restriction enzyme sites

### Yeast transformation and construction of recombinant *S. cerevisiae* strains

Transformation of plasmids for constructing engineered strains was performed using a spheroplast transformation kit (BIO 101, Vista, CA). To select transformants, *S. cerevisiae* strains were routinely cultivated aerobically at 30 °C in YNB medium (6.7 g/L yeast nitrogen base and appropriate nucleotides and amino acid). 20 g/L of ethanol was used as a carbon source for the cultivation of the SOS5 strain and 20 g/L of glucose and 1 g/L of ethanol was used for cultivation of the other strains. The *C. tropicalis PDC* gene (*CtPDC1*), *K. marxianus PDC* gene (*KmPDC1*), and *S. cerevisiae PDC* genes (*ScPDC1*, *ScPDC5*, *ScPDC6*) were expressed under *TDH3* promoter and multi-copy plasmid into the SOS5 strain. The *C. tropicalis PDC* gene was expressed under different native promoters (*TDH3*, *CYC1*, and *GPD2*) and copy number (single and multi-copy). The *PDC* expression plasmids in Table 1 were introduced into the SOS5 and BD5 strains and the engineered *S. cerevisiae* strains expressing different levels of *PDC* were constructed. Integrative *PDC* expression plasmids (p406CYC1_CtPDC1, p406GPD2_CtPDC1, and p406TDH3_CtPDC1) were digested with *Stu*I before use and integrated into the *URA3* locus. For additional expression of *L. lactis* NADH oxidase, pAUR_Llnox plasmid was transformed to the BD5_G1CtPDC1 strain. The resulting recombinant *S. cerevisiae* strains are listed in Table 1.

### Fermentation conditions

All cultures were carried out at 30 °C. Pre-cultures of yeast cells were conducted aerobically in 250-mL baffled flasks. Main flask batch cultures were conducted under microaerobic conditions in 250-mL flasks at 80 rpm. In order to prepare inoculums, engineered *S. cerevisiae* cells were cultivated for 48–72 h in 5 mL YNB medium containing 20 g/L glucose and 1 g/L ethanol. The grown cells were transferred to 100 mL YNB medium containing 20 g/L glucose and 0.5 g/L ethanol. After 24 h cultivation, the mid-exponential growing cells (OD_600_ < 3) were harvested and washed twice with double-distilled water (DDW). Cells were inoculated into the main culture at the initial concentration of 0.20 g/L. The main culture YP medium (10 g/L yeast extract and 20 g/L peptone) contains 100 g/L glucose, and YNB medium contains 90 g/L glucose and 50 mM potassium phthalate at pH 5.5 adjusted by NaOH. Ethanol 0.5 g/L was added if necessary. Batch fermentations with bioreactor were performed in 500 mL YP medium containing 100 g/L glucose using 1 L bench-top fermentor (Fermentec, Korea). For controlling aeration conditions, different ratios of mixed inlet gas (pure air, 1:1 or 1:3 of air and nitrogen gas mixture) were used at constant agitation speed of 500 rpm and air flow rate of 2 vvm. Fed-batch fermentations with bioreactor were carried out in 500 mL YP medium containing 300 g/L glucose using 1 L bench-top fermentor (Fermentec, Korea) at 30 °C. The medium pH was maintained at 5.5 with 5 N NaOH solution, and dissolved oxygen (DO) levels were monitored with O_2_ sensor (Mettler Toledo, Switzerland). The culture medium was agitated at 300–500 rpm and aerated with air flow rate of 1–2 vvm according to DO levels in medium. During the fed-batch fermentation, DO levels were kept under 2.0. The grown cells prepared from flask culture were inoculated at the initial concentration of 2.0 g/L.

### Analysis of dry cell weight and metabolites

Cell growth was monitored by optical density at 600 nm (OD_600_) using a spectrophotometer (UV-1601, Shimadzu, Japan). Dry cell weight (DCW) was calculated using a pre-determined factor of 0.20 g DCW/L/OD_600_ [36]. Glucose, glycerol, acetoin, 2,3-BD, and ethanol were analyzed by a high-performance liquid chromatography (1100 series, Agilent, CO) equipped with a Rezex ROA-organic acid column (Phenomenex, CA). Metabolites were detected by a refractive index (RI) detector. For measurement of intracellular NADH and NAD^+^, about 4 × 10^7^ of exponentially growing cells in flask batch cultivation were used. NADH and NAD^+^ concentration assays were conducted following manufacturer-provided methods (BioAssay Systems, CA).

### *In vitro* Pdc activity analysis

To prepare crude extracts, about 1 × 10^9^ mid-exponential phase cells grown on the YNB medium with 80 g/L glucose and 0.5 g/L ethanol in a flask culture were harvested and washed twice with DDW. Protease inhibitor (Roche, Switzerland) was added, and the harvested cells were lysed with Yeast Protein Extraction Reagent (Y-PER, Thermo Scientific, MA). After centrifugation for 20 min at 12,000 rpm and 4 °C, the supernatants were used to determine the Pdc activity within 3 h and diluted with DDW if necessary. The NADH oxidase activity assays were performed at 30 °C with the reaction mixture containing 40 mM imidazole hydrochloride buffer (pH 6.5), 5 mM MgCl_2_, 0.2 mM TPP, 10 U alcohol dehydrogenase from *S. cerevisiae*, 0.4 mM NADH, and 50 mM pyruvate [27]. The reactions were initiated by adding pyruvate, and a decrease of absorbance at 340 nm was measured. One unit of activity was defined as the amount of enzyme oxidizing 1 μmol NADH per minute at the corresponding reaction conditions. The protein concentration of crude extracts was determined by the Bradford method [45]. For determination of kinetic values (*K*_m_ and *V*_max_) of the Pdc enzymes, Pdc activities were measured at various concentrations of pyruvate (1.56, 3.13, 6.25, 12.5, 25.0, 50.0, 100 mM). The *K*_m_ and *V*_max_ values for Pdc were determined from Lineweaver–Burk plots of the data.

### Sample preparation for metabolite analysis

For the extraction of metabolome sampling, a fast filtration method was performed with a slight modification of the method used in a previous study [46]. Briefly, cell pellets on each conditions were collected by vacuum-filtering of 1-mL culture broth using a nylon membrane filter (0.45 μm pore size, 30 mm diameter; Whatman, Piscataway, NJ) and washed using 5 mL of distilled water at room temperature. The membrane filter and loaded cells were mixed using 10 mL of acetonitrile/water (1:1, v/v) at −20 °C. After that, the extraction mixture was immersed in liquid nitrogen. These steps were completed in less than 30 s. The extraction mixture was thawed on ice, vortexed for 3 min, and centrifuged at 16,100×*g* for 5 min at 4 °C. The supernatant was collected and vacuum-dried.

### GC/TOF MS analysis

For the GC/TOF MS analysis, the derivative metabolite samples were prepared using 5 μL of 40 mg/mL methoxyamine hydrochloride in pyridine (Sigma-Aldrich, St. Louis, MO) at 30 °C for 90 min and 45 μL of *N*-methyl-*N*-trimethylsilyl trifluoroacetamide (Fluka, Buchs, Switzerland) at 37 °C for 30 min. As retention index markers, a mixture of fatty acid methyl esters was added to the derivative samples. An Agilent 7890A FX (Agilent Technologies, Wilmington, DE) coupled with a Pegasus HT TOF MS (LECO, St. Joseph, MI) was used for metabolite analysis. A 0.5 μL of derivative samples was injected into the GC in splitless mode and separated on an RTX-5Sil MS column (30 m × 0.25 mm i.d., 0.25 μm film thickness, Restek, Bellefonte, PA) with an additional 10-m integrated guard column. The initial oven temperature was set at 50 °C for 1 min and then increased to 330 °C at a rate of 20 °C/min and held at 330 °C for 5 min. Mass spectra were recorded in the mass range of 85–500 *m/z* at an acquisition rate of ten spectra/s. The temperatures of the ion source and transfer line were set at 250 and 280 °C, respectively, and the sample ionization was performed electron impact at 70 eV.

### Data processing and statistical analysis for metabolomics analysis

For the processing of GC/TOF MS data, the software LECO Chroma TOF was used to detect peaks and to deconvolute the mass spectra. The processed data were further processed using an inhouse library, BinBase [47]. The raw metabolite data were normalized by the median of the sum of the peak intensities of all identified metabolites in each sample. The normalized data were used for multivariate and univariate statistical analysis [48], and MultiExperiment Viewer was used for the hierarchical clustering analysis (HCA).

## Additional file

**Additional file 1: Table S1.** Kinetic constants (K_m_ and V_max_) of Pdc enzymes, **Figure S1.** Batch cultivation of the BD4 strain and the BD5 strain in minimal medium, **Figure S2.** Amino acid sequence of the *C. tropicalis* pyruvate decarboxylase I, **Figure S3.** *In vitro *Pdc activities in the control and four engineered *S. cerevisiae *strains expressing *C. tropicalis* pyruvate decarboxylase gene (*CtPDC1*) differentially, **Figure S4.** Fermentation profiles of the *CtPDC1* expressing strains with glucose as a sole carbon source in minimal medium, **Figure S5.** The NADH and NAD^+^ concentrations in the BD5_G1CtPDC1_nox strain with various aeration conditions, **Figure S6.** Profiles of batch cultivations for metabolomic analysis, **Figure S7.** Principal component analysis (PCA) of intracellular metabolite profiles of BD5_G1CtPDC1 and BD5_G1CtPDC1_nox in different aerobic conditions, **Figure S8.** Hierarchical clustering analysis (HCA) of metabolite profiles of BD5_G1CtPDC1 and BD5_G1CtPDC1_nox in different aerobic conditions, **Figure S9.** Comparison of intracellular metabolites between BD5_G1CtPDC1 at aerobic condition (Ct100) and BD5_G1CtPDC1_nox at microaerobic condition (N50), **Figure S10.** Specific glucose uptake rates of the wild type D452-2 and engineered strains.
