# Supplementary material for: Enhanced production of 2,3-butanediol by engineered Saccharomyces cerevisiae through fine-tuning of pyruvate decarboxylase and NADH oxidase activities
Source: Biotechnol Biofuels. 2016 Dec 9;9:265. doi: 10.1186/s13068-016-0677-9 (PMC5148919; doi:10.1186/s13068-016-0677-9)

Table S1. Kinetic constants (K_m and V_{max}) of Pdc enzymes.

Strains	**CtPDC1**	KmPDC1	ScPDC1	ScPDC5	ScPDC6
K_m (mM)	**2.7**	7.7	4.7	9.9	8.2
V_{max} (mU/mg protein)	**107**	383	541	437	415

Figure S1. Batch cultivation of the BD4 strain (A and C) and the BD5 strain (B and D) in minimal medium containing 90 g/L glucose without ethanol (A and B) and with 0.5 g/L of ethanol (C and D). Symbols: Glucose (filled circle), dry cell weight (grey square), glycerol (blue triangle), acetoin (green inverted triangle), 2,3-BD (red star), and ethanol (open square).

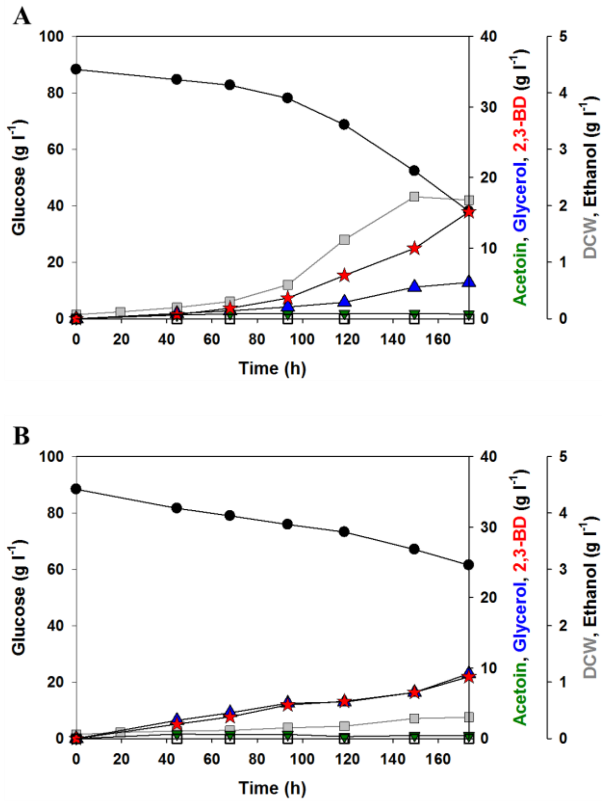

C

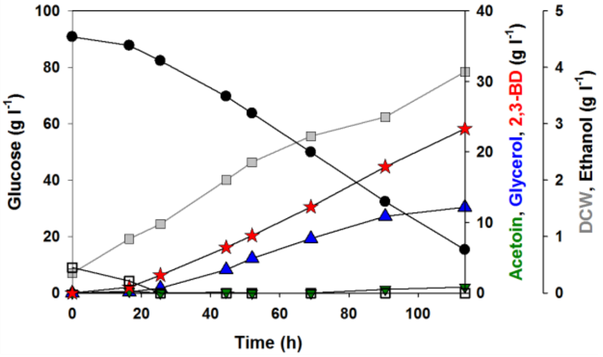

Glucose (g l-1)
Acetoin, Glycerol, 2,3-BD (g l-1)
DCW, Ethanol (g l-1)
Time (h)

D

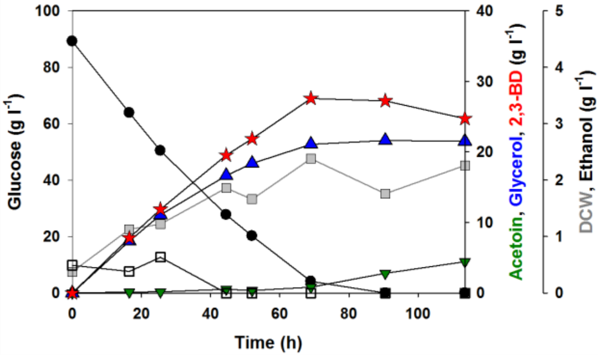

Glucose (g l-1)
Acetoin, Glycerol, 2,3-BD (g l-1)
DCW, Ethanol (g l-1)
Time (h)

Figure S2. Amino acid sequence of the *C. tropicalis* pyruvate decarboxylase I. Amino acids of Mg^{2+} and ThDP binding loop (bold and underlined) and substrate activation (bold) that are conserved throughout Pdc enzymes.

1 40
MSEITLGRFFFERLHQLQVDTVFGLPGDFNLALLDKIYEV
41 77
DGMRWAGNANELNAGYAADGYARVNPNGLAALVSTFG
78 117
VGELSLTNAIAGSYSEHVGIINLVGVPSSSAQAKQLLLHH
118 158
TLGNGDFTVFHRMFKNISQTSAFISDPNTAASEIDRCIRDA
159 198
YVYQRPVYIGLPSNLVDVKVPKSLLDKKIDLSLHPNEPES
199 239
QAEVVETVEKFISEASNPVILVDA**C**AIRHNCLKEVAELIAE
240 278
TQFPVFTTPMGKSSVDESNPRFGGVYVGSLSSPDVKEAV
279 316
ESADLVLSVGAMLSDFNTGAFSYNYKTRNVVEFHSDYT
317 355
KIRQATFPGVQMKEALQVLLKTVKKSVNPKYVPAPVPAT
356 395
KAITTPGNNDPVSQEYLWRKVSDWFQEGDVIISETG**T**SAF
396 435
GIVQSKFPKNAIGISQVLWGSIGYATGATCGAAMAAQEID
436 474
PKKRVILFTG**D**G**S**LQLTVQEISTMCKWDCYNTYLYVL**NN**
475 513
D**G**YT**I**ERLIHGEKAQYNDIQPWNNLQLLPLFNAKKYETK
514 553
RISTVGELNDLFTNKEFAVPDRIRMVEIMLPVMDAPANLV
554
AQAKQSAATNAAQE

Figure S3. *In vitro* Pdc activities in the control and four engineered *S. cerevisiae* strains expressing *C. tropicalis* pyruvate decarboxylase gene (*CtPDC1*) differentially. Cells were grown to mid-exponential phase in YNB medium containing 80 g/L glucose and 0.5 g/L ethanol and analyzed.

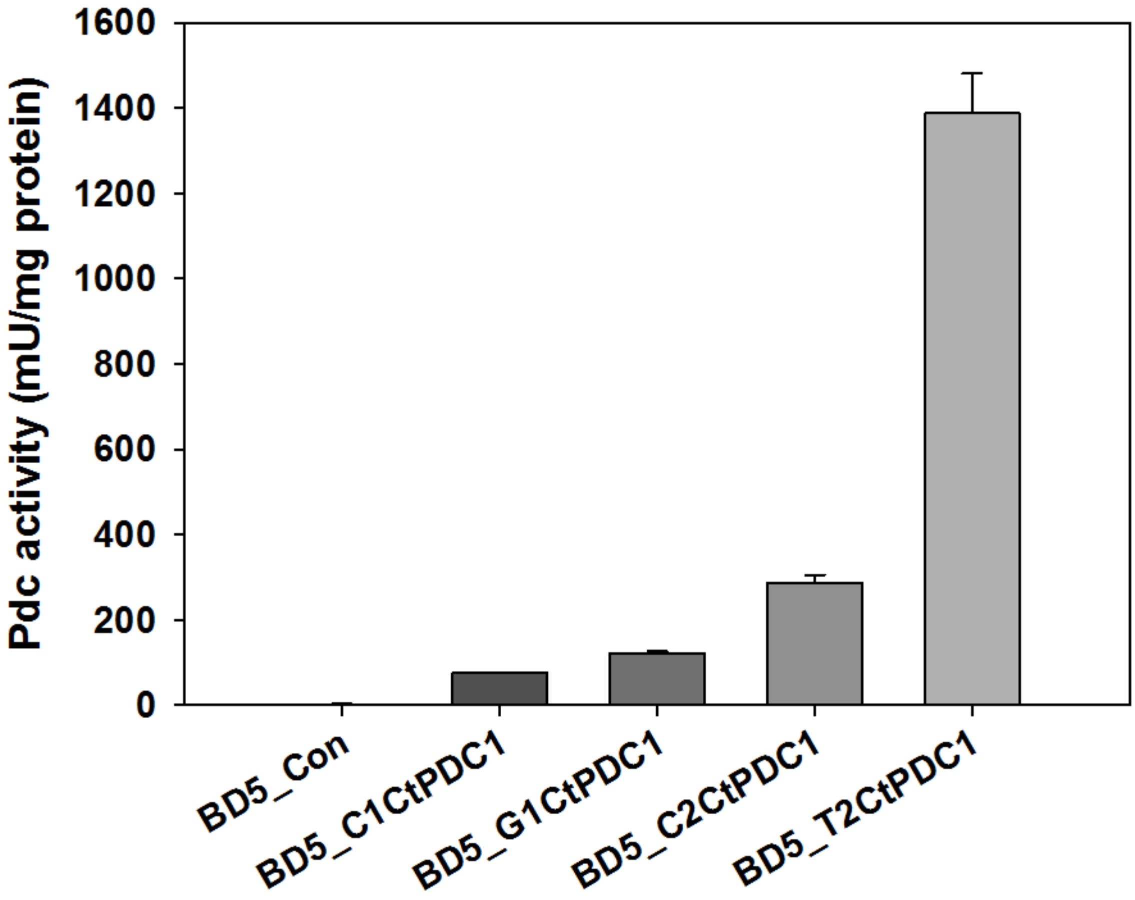

Figure S4. Fermentation profiles of the (A) BD5_Con, (B) BD5_C1CtPDC1, (C) BD5_G1CtPDC1, (D) BD5_C2CtPDC1, (E) BD5_T2CtPDC1 strains with 90 g/L glucose as a sole carbon source in minimal medium. Symbols: Glucose (filled circle), dry cell weight (grey square), glycerol (blue triangle), acetoin (green inverted triangle), 2,3-BD (red star), and ethanol (open square). Results are the averages of duplicate experiments and error bars indicate standard deviation.

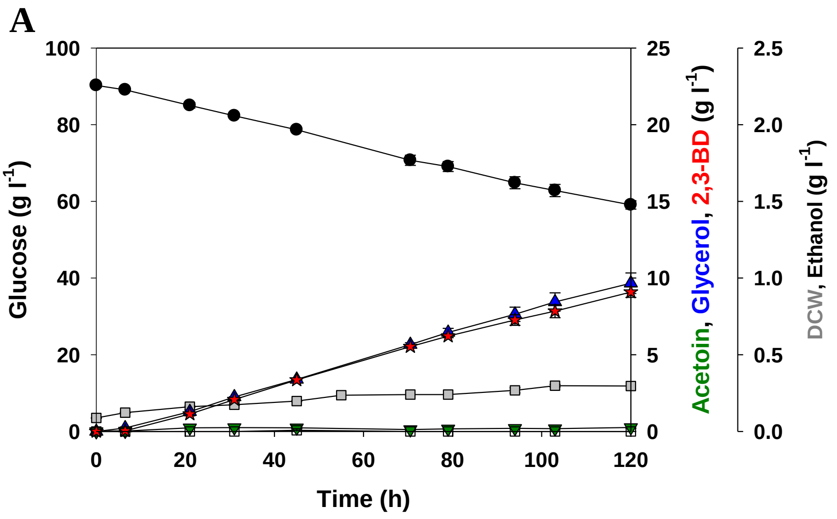

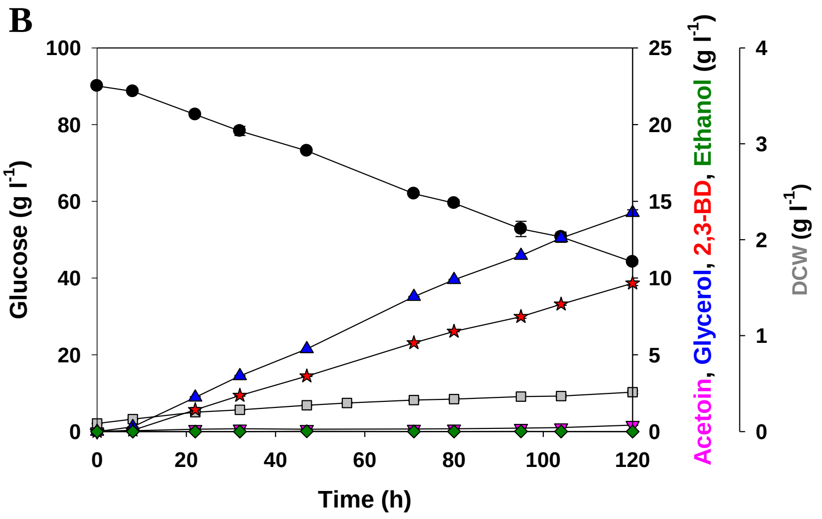

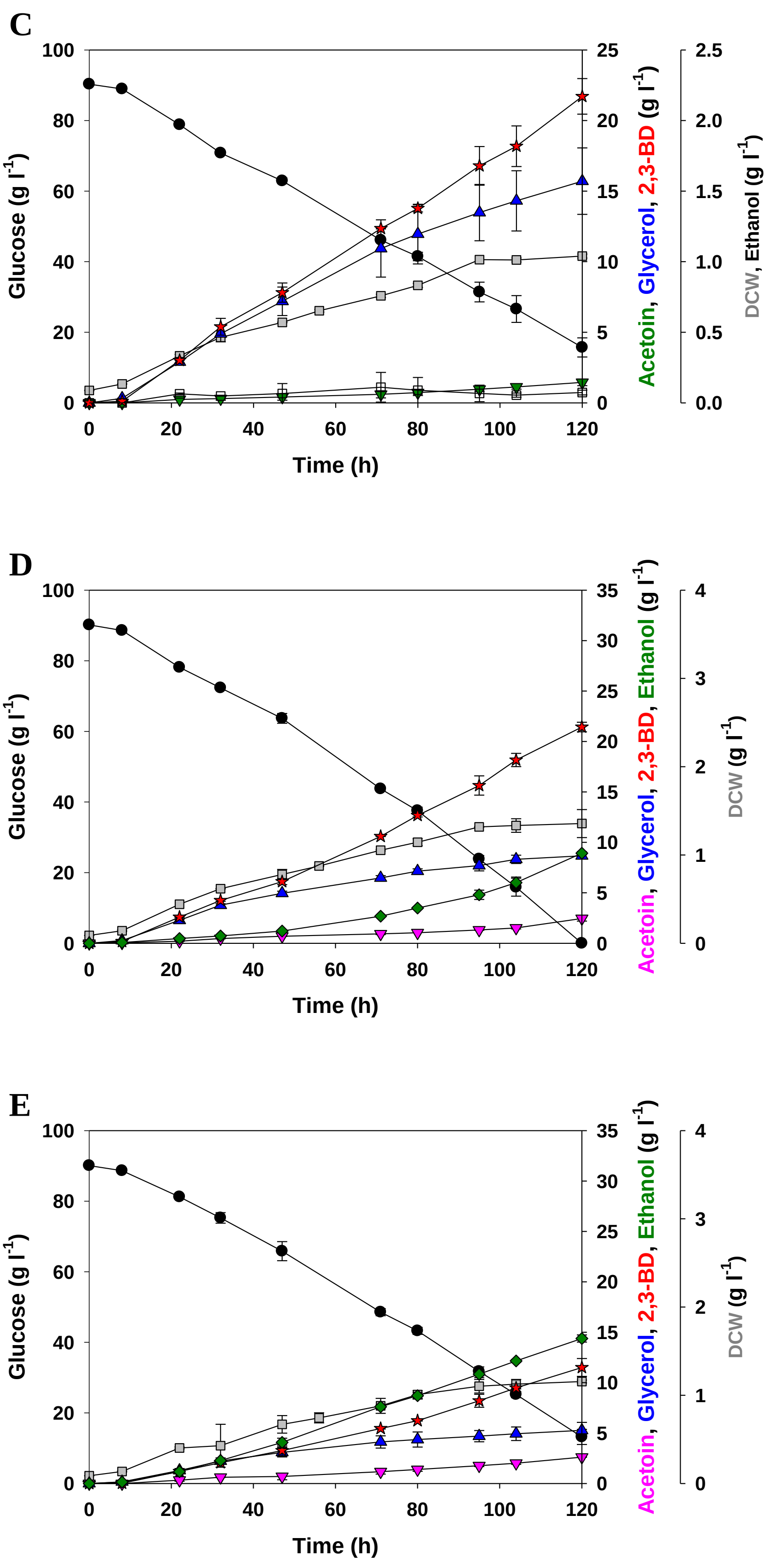
C
Glucose (g l-1)
Acetoin, Glycerol, 2,3-BD (g l-1)
DCW, Ethanol (g l-1)
Time (h)
D
Glucose (g l-1)
Acetoin, Glycerol, 2,3-BD, Ethanol (g l-1)
DCW (g l-1)
Time (h)
E
Glucose (g l-1)
Acetoin, Glycerol, 2,3-BD, Ethanol (g l-1)
DCW (g l-1)
Time (h)

Figure S5. The NADH and NAD^+ concentrations in the BD5_G1CtPDC1_nox strain with various aeration conditions. The cells from the batch cultivations at 20 h in Figure 4 were analyzed.

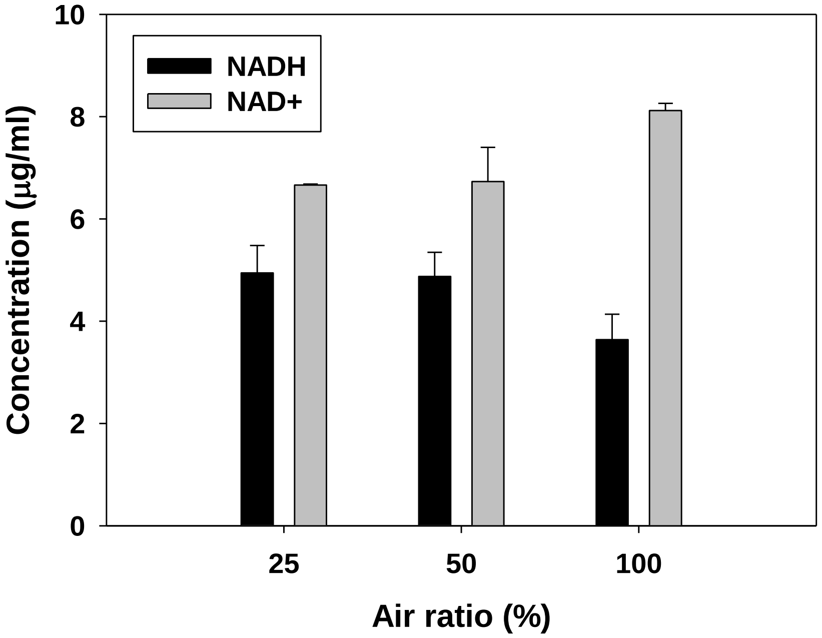

Figure S6. Profiles of batch cultivations for metabolomic analysis. The YP medium containing 100 g/L glucose was used and aeration condition was controlled by ratios of mixed inlet gas at constant agitation speed (500 rpm) and air flow rate (2 vvm). Cells were harvested at 22 h cultivation for analysis. (A) the BD5_G1CtPDC1 strain with pure air (Ct100), (B) the BD5_G1CtPDC1_nox strain with 1:1 mixture of air and nitrogen gas (N50), (C) the BD5_G1CtPDC1_nox strain with pure air (N100).

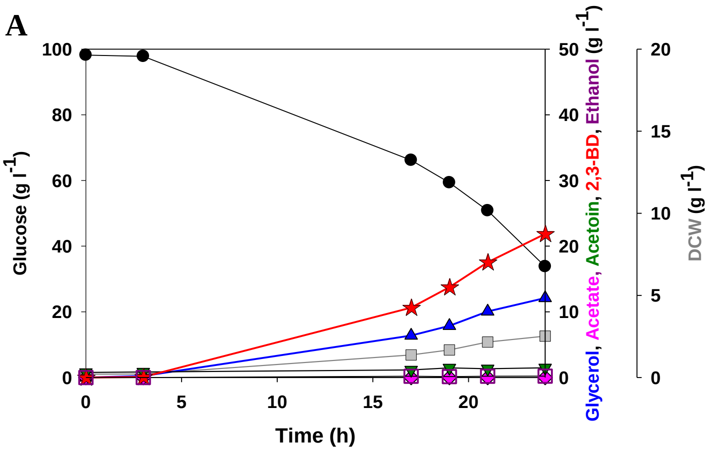

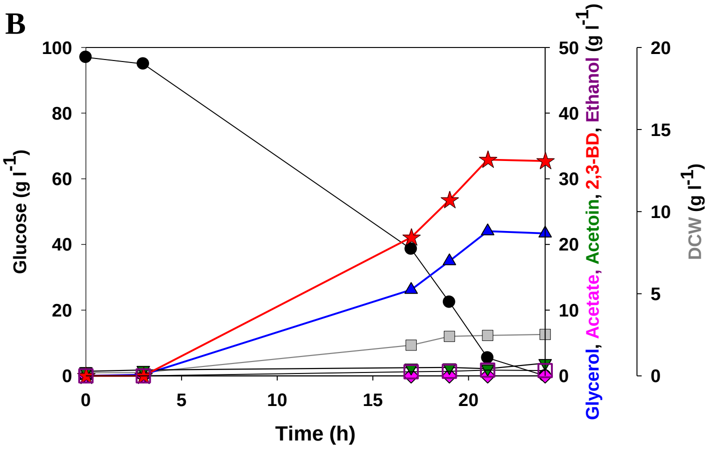

C

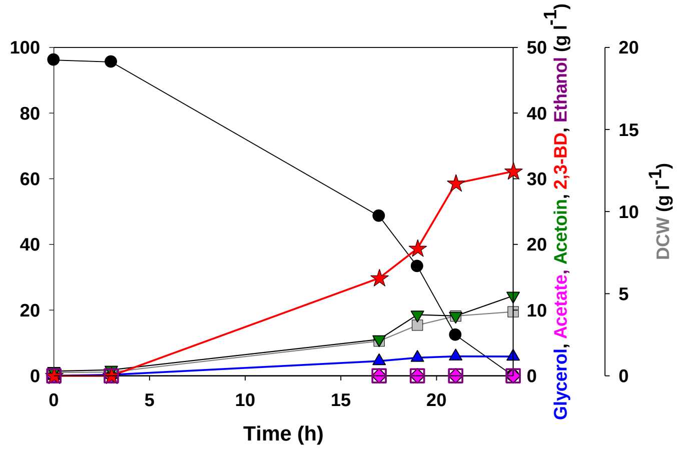

Glucose (g l-1)
100
80
60
40
20
0
0
5
10
15
20
Time (h)
Glycerol, Acetate, Acetoin, 2,3-BD, Ethanol (g l-1)
50
40
30
20
10
0
DCW (g l-1)
20
15
10
5
0

Figure S7. Principal component analysis (PCA) of intracellular metabolite profiles of BD5_G1CtPDC1 and BD5_G1CtPDC1_nox in different aerobic conditions.

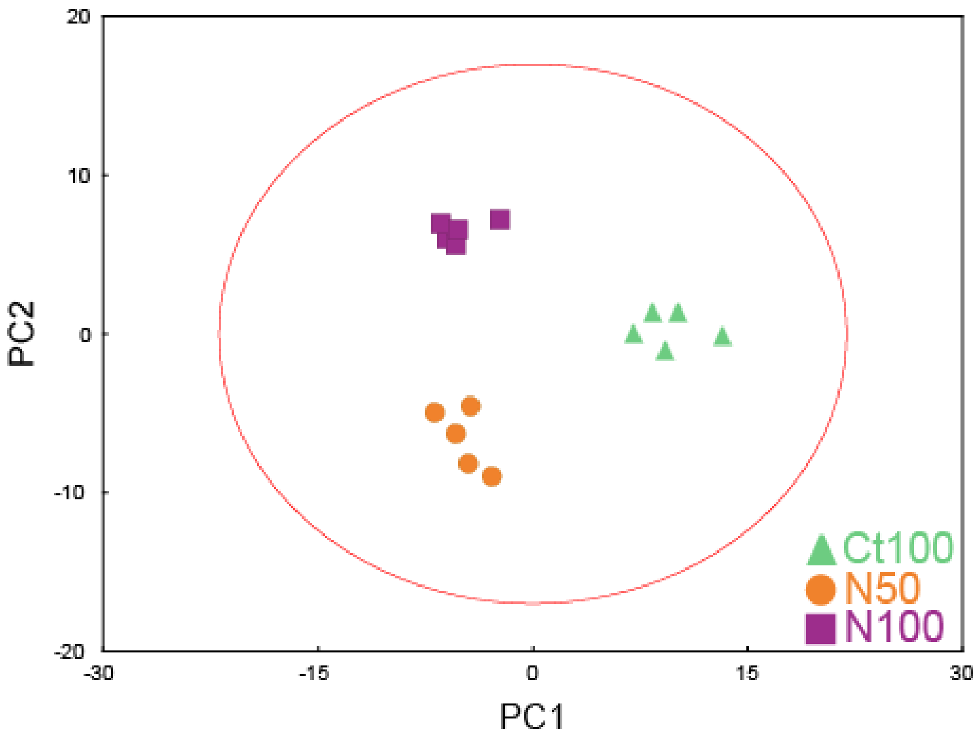

Figure S8. Hierarchical clustering analysis (HCA) of metabolite profiles of BD5_G1CtPDC1 and BD5_G1CtPDC1_nox in different aerobic conditions. Red-bars represent the increased metabolites and blue-bars represent the decreased metabolites.

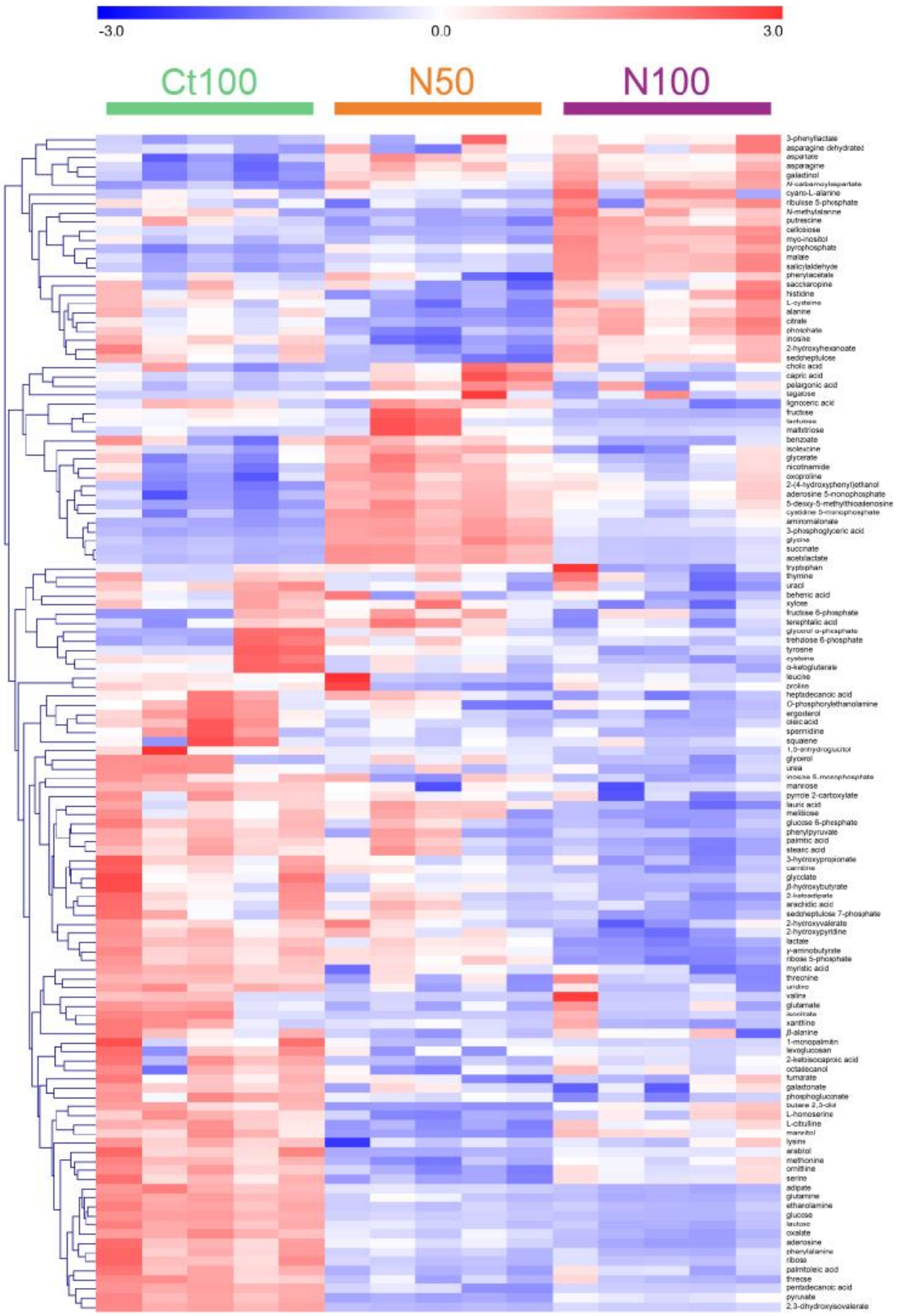

Figure S9. Comparison of intracellular metabolites between BD5_G1CtPDC1 at aerobic condition (Ct100) and BD5_G1CtPDC1_nox at microaerobic condition (N50). Compounds with italic letters indicates identified and quantified metabolites. The underlined characters are increased metabolites in Ct100 condition, and the metabolites with asterisk (*) are increased metabolites in N50 condition. The other metabolites without underlined character and asterisk are not statistically significant (p > 0.05).

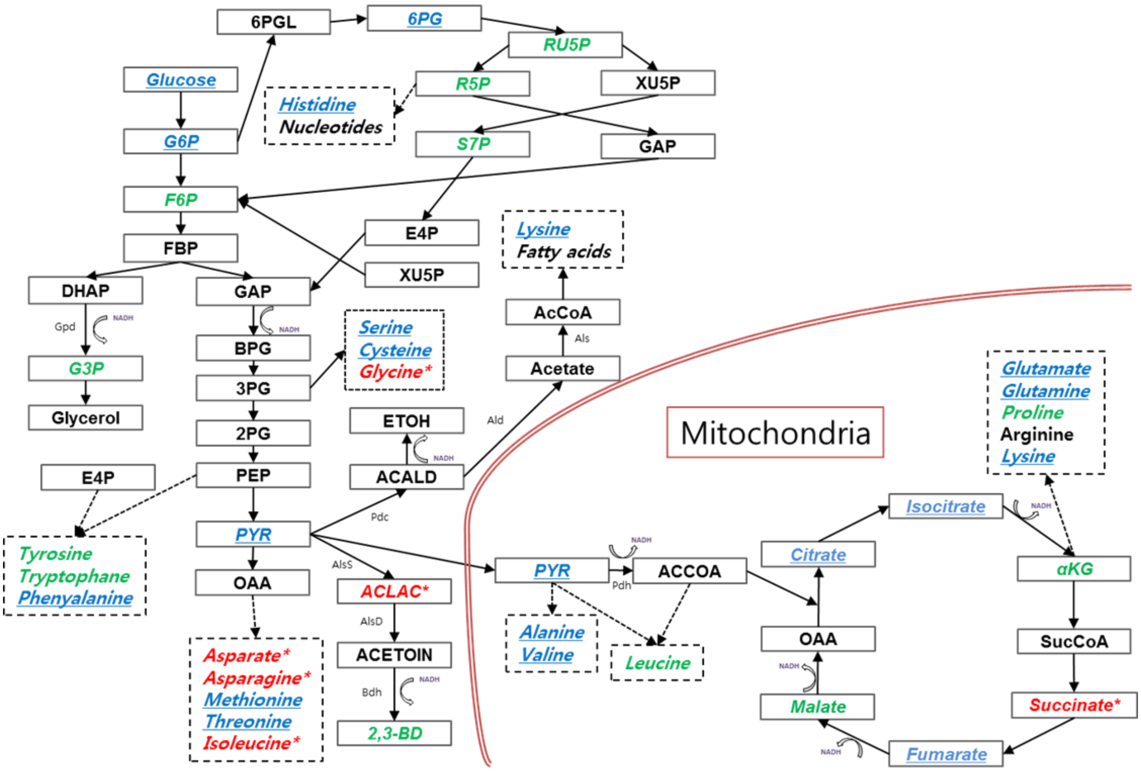

Figure S10. Specific glucose uptake rates of the wild type D452-2 and engineered strains. To determine the specific glucose uptake rate, the initial OD10 of cells were cultivated in YP medium with 100 g/L glucose. The amounts of glucose consumed in 9 h cultivation were used for calculating specific glucose uptake rates.

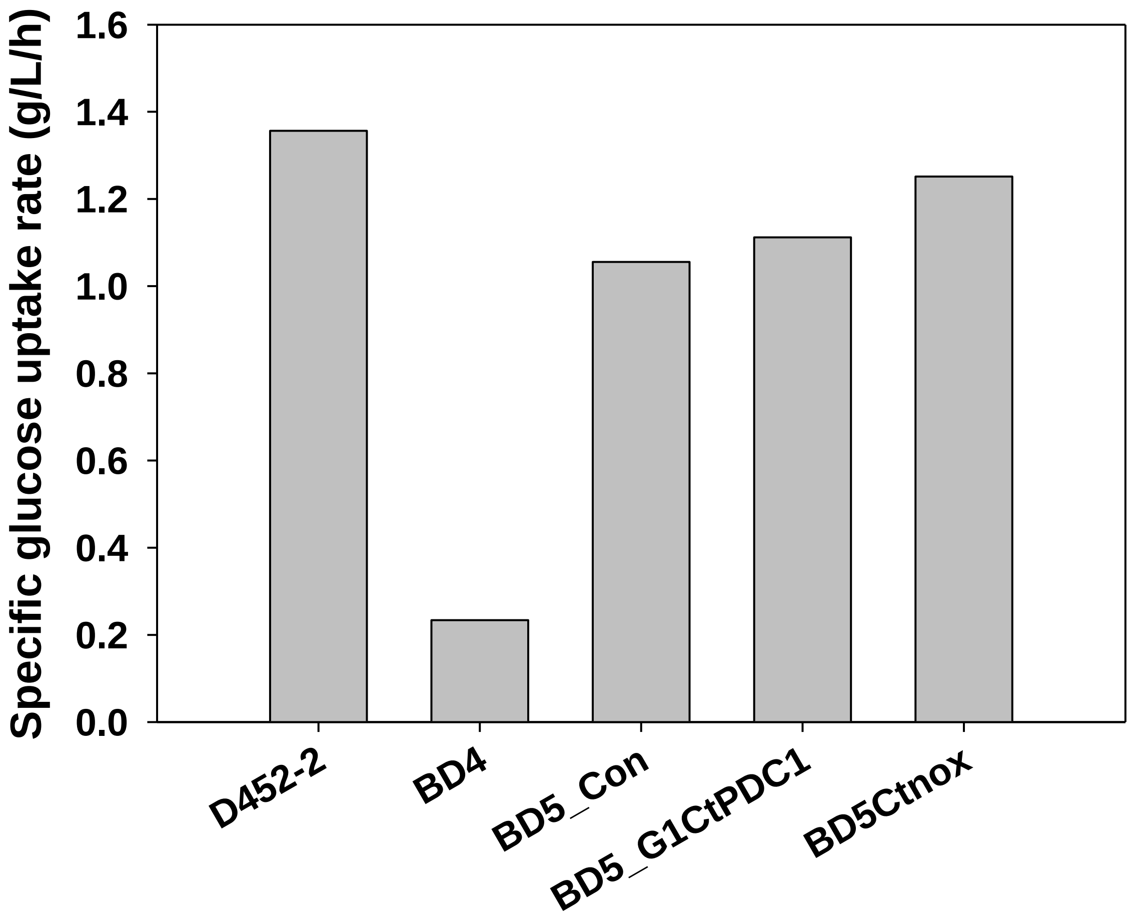

Supplement: Supplementary file 1 — Additional file 1: Table S1. Kinetic constants (Km and Vmax) of Pdc enzymes, Figure S1. Batch cultivation of the BD4 strain and the BD5 strain in minimal medium, Figure S2. Amino acid sequence of the C. tropicalis pyruvate decarboxylase I, Figure S3. In vitro Pdc activities in the control and four engineered S. cerevisiae strains expressing C. tropicalis pyruvate decarboxylase gene (CtPDC1) differentially, Figure S4. Fermentation profiles of the CtPDC1 expressing strains with glucose as a sole carbon source in minimal medium, Figure S5. The NADH and NAD+ concentrations in the BD5_G1CtPDC1_nox strain with various aeration conditions, Figure S6. Profiles of batch cultivations for metabolomic analysis, Figure S7. Principal component analysis (PCA) of intracellular metabolite profiles of BD5_G1CtPDC1 and BD5_G1CtPDC1_nox in different aerobic conditions, Figure S8. Hierarchical clustering analysis (HCA) of metabolite profiles of BD5_G1CtPDC1 and BD5_G1CtPDC1_nox in different aerobic conditions, Figure S9. Comparison of intracellular metabolites between BD5_G1CtPDC1 at aerobic condition (Ct100) and BD5_G1CtPDC1_nox at microaerobic condition (N50), Figure S10. Specific glucose uptake rates of the wild type D452-2 and engineered strains. [file 13068_2016_677_MOESM1_ESM.pdf]
